# The Role of Ferulic Acid in Selected Malignant Neoplasms

**DOI:** 10.3390/molecules30051018

**Published:** 2025-02-23

**Authors:** Anna Markowska, Janina Markowska, Joanna Stanisławiak-Rudowicz, Katarzyna Kozak, Otton Krzysztof Roubinek, Magdalena Jasińska

**Affiliations:** 1 Department of Perinatology, Poznań University of Medical Science, 60-535 Poznań, Poland; annamarkowska@vp.pl; 2 Gynecologic Oncology Center Poznań, Poznańska 58A, 60-850 Poznań, Poland; jmarkmed@poczta.onet.pl; 3 Department of Gynecologic Oncology, Poznań University of Medical Sciences, Szamarzewskiego 84, 60-514 Poznań, Poland; stanisl@interia.pl; 4 Łukasiewicz Research Network—Industrial Chemistry Institute, Rydygiera 8, 01-793 Warszawa, Poland; katarzyna.kozak@ichp.lukasiewicz.gov.pl; 5 Faculty of Chemical and Process Engineering, Warsaw University of Technology, Waryńskiego 1, 00-645 Warszawa, Poland; magdalena.jasinska@pw.edu.pl

**Keywords:** ferulic acid (FA), flavonoids, anticancer activity, breast cancer, cervical cancer, prostate cancer, colon cancer

## Abstract

Ferulic acid (FA) is a polyphenol that is found in plants and fruits. It has a wide range of anticancer properties, including participating in cell apoptosis, inhibiting invasion and angiogenesis, and acting synergistically with standard cytostatic agents in malignant tumors. A range of molecular mechanisms are involved in anticancer activity and include the following ones: activation of cell-cycle-related proteins and enzymes such as p53, p21, Bax, and pro-caspases 3 and 9, reduction of cyclin D1 and E, proapoptotic Bcl-2, MMP-9, and NF-kV, which decrease VEGF, leading to cell cycle arrest at G0/G1 phase and death of cancer cells. Other mechanisms inhibit several pathways: PI3K/AKT/mTOR, Notch, and Wnt, which are associated with downregulation of proliferation, invasion, metastasis, and angiogenesis. FA can induce activation of ROS, leading to DNA damage in cancer cells. In vitro and in vivo studies have demonstrated the significant antitumor activity of FA in breast cancer, particularly when used in combination with cytostatic agents. In vitro studies on cervical cancer cell lines have reported similar anticancer activity of FA. This includes inhibition of cell proliferation and induction of apoptosis by downregulating antiapoptotic proteins. A case-control study conducted in Italy found that men with histologically confirmed prostate cancer had notably lower levels of FA compared to controls. Molecular in vitro studies have suggested that FA may have various effects on the signaling pathways linked to a reduction in the risk of prostate cancer, and it may act in synergy with δ-tocotrienol, which is a derivative of vitamin E. In vivo and in vitro studies on colorectal cancer have demonstrated the effects of FA on the early development of this cancer—inhibition of abnormal crypt foci (ACF-aberrant crypt foci), as well as the reduction in cancer cell viability and apoptosis through molecular changes, mainly a decrease in EGFR expression. The poor water solubility of FA makes it an attractive candidate for use as nanoparticles.

## 1. Introduction

**Figure 1:**
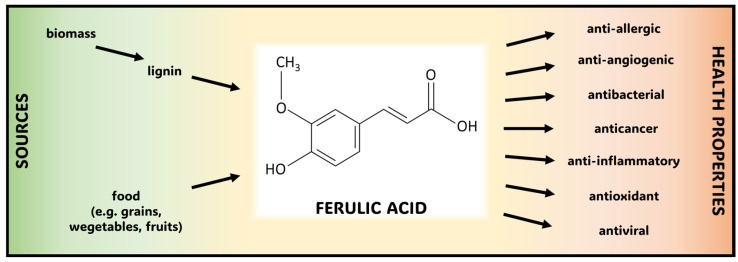
Chemical structural formula for the FA molecule with various sources and health properties.

Ferulic acid (FA)—4-hydroxy-3-methoxycinnamic acid—is an organic chemical compound belonging to the phenolic acids subgroup of polyphenolic compounds. It was first isolated from the plant *Ferula foetida*. It is commonly found in fruits, vegetables, cereals, seeds, and herbs [1,2,3,4]. FA has various pharmacological activities: antioxidant, anti-inflammatory, antiangiogenic, antiallergic, antimicrobial and antiviral, neuroprotective, and anticancer [3,5,6,7,8]. Polyphenols encountered in plants and in particular in fruits exhibit anticancer properties. These compounds can be delivered into human body in the form of food, i.e., by the application of an adequate diet. Polyphenols and flavonoids are currently utilized in conceptual works concerning oncological therapies [9]. Interestingly, in order to obtain and produce polyphenols including FA, natural organic compounds can be successfully applied. For instance, biodegradation of lignocellulose or even lignin catalyzed by a carefully selected and extracted enzymes can be recommended [10,11]. In case of FA, a renewable source of organic carbon in the form of agriculture waste can be proposed. In particular, wastes received from processing of plant raw materials which are not further used in production of the first-generation biofuels can provide an efficient method in the nearest future. In work of Iram et al. [12], the method of a treatment of a plant waste in the production of first-generation bioethanol has been presented and described in detail. In this work [12], it was also shown that the same plant waste can be simultaneously used for production of second-generation ethanol, confirming that a single source can be utilized as an efficient source of two different products. In conclusion, a new approach in which lignin is processed into polyphenols with an application of biotechnological methods can be proposed and applied. A chemical structural formula of an FA compound with health properties and various sources is presented in Figure 1.

Malignant tumors are an increasingly common threat to human health and life. Global statistics on the incidence and mortality rates of 36 different types of malignant tumors in various locations in 185 countries (Globocan) are alarming. In 2020, there were 19.3 million new cases of malignant tumors and 10.5 million deaths due to cancer worldwide. The global cancer burden is estimated to reach 28.4 million in 2040, a 47% increase in incidence compared to in 2020 [13]. Thanks to earlier cancer detection, mortality from some malignancies is decreasing, but the incidence of the most common cancers, including female breast cancer and prostate cancer, is steadily increasing [14]. Several treatments are available for malignant tumors, including surgery, chemotherapy, targeted therapy, and immunotherapy. Recent studies have shown the positive effects of natural plant substances, such as ferulic acid (FA), in preventing the development of various types of cancer, including breast, colon, prostate, and cervical cancers. FA has been found to have a multidirectional approach in its anticancer effects (Table 1), as described in several publications [15,16,17,18,19,20]:

- Involvement in apoptosis of tumor cells;
- Disruption of the cell cycle by arresting it in the G0/G1 phase;
- Inhibition of migration, invasion, and angiogenesis;
- Synergistic effect with standard cytostatic used;
- Reduction in side effects of oncology treatment.

**Figure 2:**
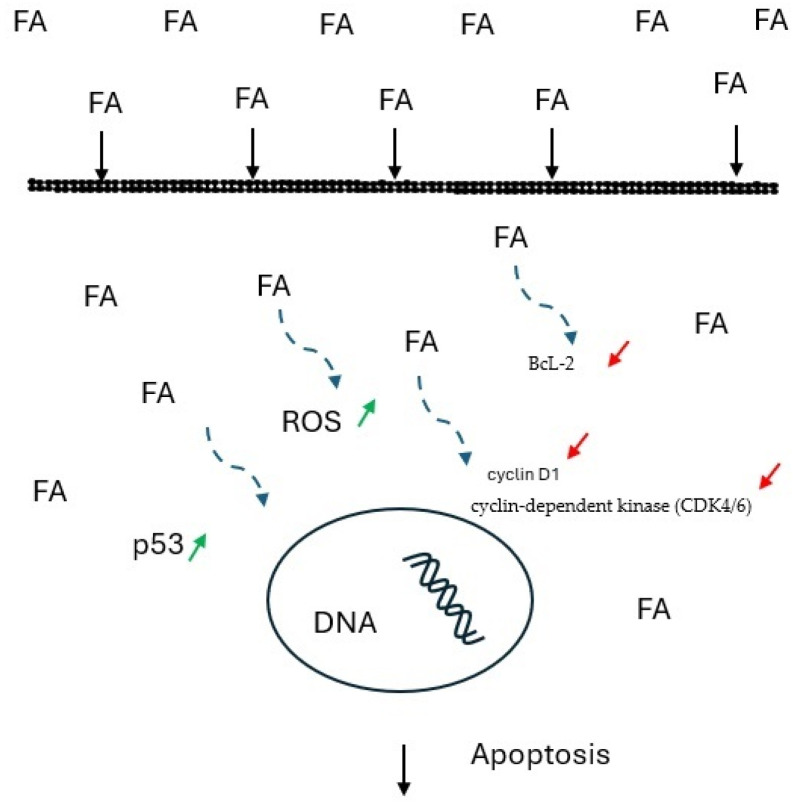
Simplified scheme of FA’s impact on the DNA of cancer cells.

FA causes cell proliferation and apoptosis inhibition by increasing the expression of tumor suppressor p53, decreasing the expression of cyclin D1 and cyclin-dependent kinase (CDK4/6), and blocking the expression of the antiapoptotic protein BcL-2. FA affects cell mitochondrial apoptosis by inducing the production of intracellular reactive oxygen species (ROS), leading to cell DNA damage, genotoxic stress, and subsequent cell death. The suppression of PI3K/AKT/mTOR pathway signaling inhibits tumor migration, invasion, and angiogenesis. In addition, FA inhibits angiogenesis by decreasing the expression of VEGF mRNA and protein and by fibroblast growth factor-FGF1. Increased expression of the effector enzyme caspase 3 and inhibition of the nuclear transcription factor NF-kβ associated with antitumor apoptosis and inhibition of proliferation have also been described. The mechanism of FA action and its impact on cancer cells, as described above, is depicted in simple terms in Figure 2, based on exemplary mechanisms presented in several studies [6,15,18,21,22].

## 2. Breast Cancer

**Figure 3:**
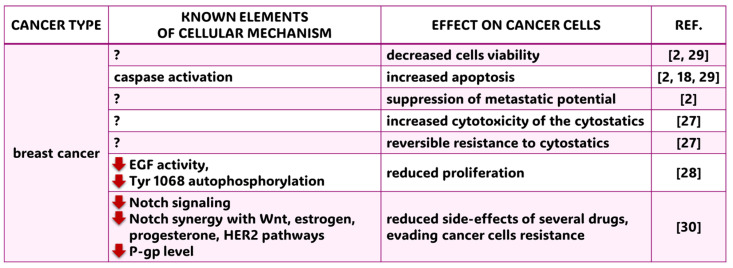
Known cellular mechanisms and effects of FA application on breast cancer. ?—elements of cellular mechanisms have not been revealed yet; red arrow heading down—diminished cellular effect; REF.—references: Zhang et al. (2016) [2], Yang et al. (2015) [18], Meirelles et al. (2023) [27], Sudhagar et al. (2018) [28], Rezaei et al. (2019) [29], Helmy et al. (2022) [30].

Breast cancer is the most commonly diagnosed cancer in the world. In 2022, there were 2.3 million new cases and 666,000 deaths, and the number of cases is projected to rise to 3 million, with deaths expected to reach 1 million by 2040. The cancer is a threat to women’s health worldwide [13,23]. The efficacy of common medications is often hindered by both drug resistance and treatment side effects [24]. Traditional Chinese medicine employs ferulic acid and other pharmaceuticals to assist in standard breast cancer treatment. These agents improve the tumor cell microenvironment, regulate epithelial−mesenchymal transition (EMT) and inhibit proliferation and metastasis [25]. Alotabi et al. [26] used a molecular method (qRT-PCR) and flow cytometry on breast cancer cell lines (MDA-MB-231) to detect increased expression of the pro-apoptotic genes BAX and p53 and decreased expression of the antiapoptotic gene BCL-2. According to the authors, the observed induction of apoptosis depends mainly on p53, and the compounds studied, especially ferulic acid, may be an essential anticancer agent. Zhang et al. [2] conducted research on the potential anticancer properties of ferulic acid. Their findings revealed that ferulic acid can reduce cancer cell viability, increase apoptosis and suppress metastatic potential both in vitro and in a xenograft model using MDA-MB-21 cells in mice. These results suggest that ferulic acid has the potential to be used as an effective therapeutic agent for the treatment of breast cancer. In other studies on four breast cancer cell lines, including the MDA-MB-231 and MCF-7 lines, FA showed positive anticancer effects in combination with conventionally used cytostatics: cisplatin, paclitaxel, doxorubicin, and tamoxifen. Increased cytotoxicity of the drugs and reversible resistance to cytostatics were observed [27]. Sudhagar et al. [28] obtained similar tumor-suppressive results. Ferulic acid reduced breast cancer cell proliferation by inhibiting EGF activity and decreasing Tyr 1068 autophosphorylation in vitro. The results of this study may help develop new anti-EGFR nanoparticles. According to Rezaei et al. [29], ferulic acid, as it is poorly water-soluble, has limited use in the food and pharmaceutical industries. Encapsulating FA in cyclodextrin nanoshells (CD-NSs) and applying this form to breast cancer cell lines (MCF7 and 471) significantly reduced cancer cell viability and increased apoptosis compared to pure FA. Thus, CO-NSs proved to be a suitable delivery system for active, poorly soluble substances to cancer cells. Studies on nanoparticles by other authors have shown that using silica nanoparticles containing bioactive compounds, including FA with platinum conjugates, is effective. Nanoparticles in vitro and in vivo in BALB mice induced apoptosis via caspase activation [18]. In a study by Helmy et al. [30], the anticancer activity of nanoparticles containing ferulic acid derivatives (transferulic acid, doxycycline lactic acid polyacid, and Dox/Fa-PLGA-TFA) proved effective in breast cancer. The nanoparticle inhibited Notch and Wnt signaling associated with cell survival and apoptosis and downregulated P-glycoprotein (P-gp), which belongs to multidrug-resistant proteins. The use of FA nanocarriers consisting of multi-walled carbon nanotubes (CNTs) with two natural anticancer agents, FA and diosgenin (a plant containing steroidal saponins), has proven to be a system that delivers active natural prodrugs to MCF-7 breast cancer cells [31]. The findings on FA and the search for new nanocarriers for its delivery to cancer cells may advance additional therapy for this cancer. A summary of current knowledge on the molecular mechanisms and effects of ferulic acid application on breast cancer is presented in the Figure 3.

## 3. Cervical Cancer

**Figure 4:**
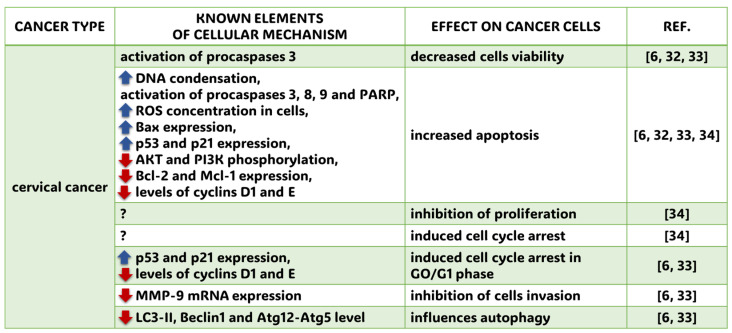
Known cellular mechanisms and effects of FA application on cervical cancer. ?—elements of cellular mechanisms have not been revealed yet; blue arrow heading up—enhanced cellular effect; red arrow heading down—diminished cellular effect; REF.—references: Gupta et al. (2021) [6], Gao et al. (2018) [32], Luo et al. (2020) [33], Wang et al. (2022) [34].

Cervical cancer is the fourth most commonly diagnosed cancer in women. In 2022, more than 660,000 women worldwide were affected, and there were more than 350,000 deaths [13]. Chronic infection of the cervix with high-oncogenic human papillomavirus—HPV—is responsible for the main etiological factor. In addition, lifestyle influences the development and course of this cancer. FA inhibits the development of cervical cancer, as shown by in vitro studies on HeLa and Caski cell lines. FA significantly reduced cell viability (*p* < 0.05) and significantly increased apoptosis (*p* < 0.05) in Caski cells. The molecular mechanism was activation of procaspases 3, 8, and 9, and a dose-dependent decrease in AKT and PI3K phosphorylation was observed [32]. Gao et al. [33] reported reduced viability using FA in 88.3% of HeLA cells and 85.4% of Caski cells. The molecular mechanism was thought to be a reduction in mRNA expression of matrix metalloproteinase 9 (MMP-9). FA-induced cell cycle arrest in GO/G1 phase occurs in a dose-dependent manner significantly (*p* < 0.05). In addition, FA induced the expression of cellular proteins p53 and p21 and decreased the levels of cyclins D and E. Gupta et al. [6] described similar changes. The authors also found decreased levels of the antiapoptotic protein Bcl-2. Wang et al. [34] studied the effects of an FA derivative, FA 30. This substance inhibited cell proliferation in the HeLa line by inducing apoptosis and induced cell cycle arrest by affecting the increase of ROS concentration in cells. The results of the study are consistent with those described in breast cancer cells and reinforce the suggestion of using FA as a supplemental therapy. A summary of up-to-date information on the molecular mechanisms and effects of ferulic acid application on cervical cancer is depicted in the Figure 4.

## 4. Prostate Cancer

**Figure 5:**
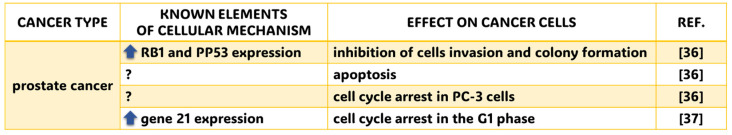
Known cellular mechanisms and effects of FA application on prostate cancer. ?—elements of cellular mechanisms have not been revealed yet; blue arrow heading up—enhanced cellular effect; REF.—references: Eroglu et al. (2015) [36], Eitsuka et al. (2014) [37].

Prostate cancer is the most common cancer in men. According to the Globocan world statistics, there were 1.5 million new cancer cases and 397,000 deaths in 2022. In more than half of the world’s countries included in the statistics (112/180), it is the most common malignant tumor [13]. Russo et al. [35] conducted a case-control study in Sicily (mid-Italy) on the effects of FA consumption and another flavonoid, caffeic acid, on the risk of developing prostate cancer. They studied 118 cases of histologically verified prostate cancer and 222 men in the control group using questionnaires and multivariate logistic regression. It was shown that those with prostate cancer had significantly lower FA levels compared to the control group (2.80 mg/day vs. 4.04 mg/day; *p* < 0.01). Similar correlations were shown in the level of caffeic acid (2.28 mg/day vs. 2.76 mg/day; *p* < 0.05). A high intake of ferulic acid and caffeic acid may be associated with a reduced risk of prostate cancer. Eroglu et al. [36] examined the effect of FA on prostate cancer cell lines (PC-3 and LNCaP) by analyzing 84 key genes involved in cell cycle regulation and apoptosis. FA increased the expression of some genes, including those related to cell cycle inhibition, including RB1 and PP53. In addition, FA in both cell lines inhibited invasion and colony formation. At the same time, differential effects of FA on cell lines were found; it leads to cell cycle arrest in PC-3 cells and causes apoptosis in the LNCaP cell line. This result indicates possible different molecular pathways involved in the development of prostate cancer. Furthermore, the research unveiled a promising synergy. FA, when combined with δ—tocotrienol (a vitamin E derivative), demonstrated a potent effect in an in vitro test on the DV-145 cell line. This combination led to cell cycle arrest in the G1 phase by significantly increasing the expression of gene 21 in these cells [37]. The authors suggest that combining tocotrienol δ fractions with FA may be a strategy for prostate cancer prevention and even treatment. A summary of actual data on the molecular mechanisms and effects of ferulic acid application on prostate cancer is presented in the Figure 5.

## 5. Colorectal Cancer

**Figure 6:**
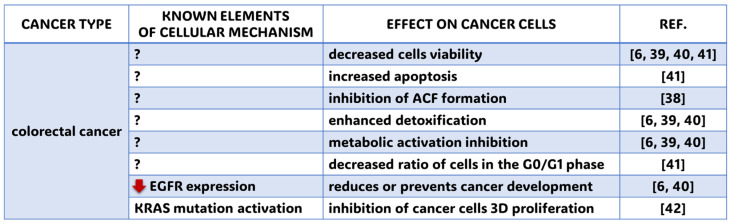
Known cellular mechanisms and effects of FA application on colorectal cancer. ?—elements of cellular mechanisms have not been revealed yet; red arrow heading down—diminished cellular effect; REF.—references: Gupta et al. (2021) [6], Kawabata et al. (2000) [38], Janicke et al. (2005) [39], Roy et al. (2016) [40], Rosa et al. (2018) [41], Sawata et al. (2019) [42].

In 2022, more than 1.9 million new cases of colorectal cancer were reported worldwide. In the same year, there were approximately 904,000 deaths due to this cancer. This statistic indicates that 1 in 10 cancer cases is colorectal cancer [13]. It is the third most common cancer in terms of incidence and second in terms of mortality worldwide. Risk factors are primarily a shift to a diet rich in animal products, a sedentary lifestyle with decreased physical activity, overweight, and obesity. Additional risk factors include smoking and excessive consumption of alcohol, red meat, and processed foods. In contrast, meals rich in whole grains, fiber, or dairy products appear to reduce the risk of the disease [13]. Kawabata et al. [38] noted in their work the therapeutic activity of FA against azoxymethane (AOM), a carcinogen in rats’ colons. The study was conducted at various concentrations and noted that a concentration of 15 mg/kg inhibited the formation of aberrant crypt foci (ACFs), the earliest developing precursors of epithelial neoplasia. One of the mechanisms of FA against carcinogenesis within colon cells is inhibiting metabolic activation and enhancing detoxification [6,39]. The effect of FA (100–250 μg/mL) on a colorectal cancer cell line (HCT-15), as well as on epidermal growth factor receptor (EGFR) gene expression, was also investigated. It was noted that FA administration decreased HCT-15 cell viability in a concentration-dependent manner (IC50 154 μg/mL). In addition, FA (175 μg/mL) decreased gene expression for EGFR by 4.28-fold compared to the control group and showed the ability to bind to the EGFR gene at the molecular level in silico [6,40]. Rosa et al. [41] evaluated the effect of FA on cell viability and the apoptosis rate in human adenocarcinoma cells. FA (5 μM) promoted an approximately 63% decrease in cell viability and caused a decrease in the ratio of cells in the G0/G1 phase (10 and 100 µM) of the cell cycle, as well as an increase in the percentage of apoptotic cells. The scientific literature reports that when added to caffeic acid, resveratrol and its derivatives inhibit the three-dimensional (3D) proliferation of the colorectal cancer cell line HCT116, with an activating KRAS mutation. Resveratrol bound to ferulic acid also inhibits the 3D proliferation of HCT116 cells more potently than resveratrol alone [42]. The authors suggest that combining tocotrienol δ fractions with FA may be a strategy for prostate cancer prevention and even treatment. Actual information on the molecular mechanisms and effects of ferulic acid application on colorectal cancer is summarized in the Figure 6 and Table 1.

**Table 1:** Effects of ferulic acid application on different types of cancer cells in in vitro and in vivo models. REF.—references.

Cancer Type	Effect of FA Application	Model	REF.
**Breast cancer**	decreased cancer cell viability, increased apoptosis, suppression of metastatic potential	In vitro and xenograft model MDA-MB-21 cells in mice	[2]
	increased cytotoxicity of the cytostatics reversible resistance to cytostatics	In vitro MDA-MB-231, MCF-7, MDA-MB-468, and BT-20 cell lines	[27]
	EGF activity inhibition, decreased Tyr 1068 autophosphorylation, reduced breast cancer cell proliferation	In vitro MCF7 cell line	[28]
	decreased cancer cell viability increased apoptosis	In vitro MCF7 and 471 cell lines	[29]
	caspase activation increased apoptosis	In vitro and in vivo BALB mice	[18]
	Notch and Wnt signaling inhibition P-gp downregulation	In vivo Female Sprague–Dawley rats	[30]
**Cervical cancer**	procaspases 3, 8, and 9 activation, decreased AKT and PI3K phosphorylation, decreased cancer cell viability, increased apoptosis	In vitro HeLa and Caski cell lines	[32]
	reduced MMP-9 mRNA expression, decreased cancer cell viability, induced cell cycle arrest in GO/G1 phase, induced p53 and p21 cellular expression, decreased levels of cyclins D and E	In vitro HeLa and Caski cell lines	[6,33]
	decreased Bcl-2 level	In vitro HeLa and Caski cell lines	[6]
	cell proliferation inhibition, increased apoptosis, induced cell cycle arrest, increased ROS concentration in cells	In vitro HeLa cell line	[34]
**Prostate cancer**	increased expression of RB1 and PP53 invasion and colony formation of cells inhibition	In vitro PC-3 and LNCaP cell lines	[36]
	cell cycle arrest in PC-3 cells apoptosis of cells	In vitro LNCaP cell line	[36]
	increased expression of gene 21 cell cycle arrest in the G1 phase	In vitro DV-145 cell line	[37]
**Colorectal cancer**	inhibition of ACF formation	In vivo F334 rats	[38]
	metabolic activation inhibition, enhanced detoxification, decreased cancer cell viability, decreased EGFR expression	In vitro HCT-15 cell line	[6,39,40]
	decreased cancer cell viability, decreased ratio of cells in the G0/G1 phase, increased apoptosis	In vitro human adenocarcinoma cells	[41]
	cancer cells 3D proliferation inhibition KRAS mutation activation	In vitro HCT116 cell line	[42]

## 6. Pros and Cons of Ferulic Acid Application in Cancer Treatment

Ferulic acid exhibits many properties that are indisputably beneficial for human health. Most importantly, it reduces the oxidative stress and inflammation rates associated with cancer promotion and progression. By modulating several signaling pathways, FA appears to be useful in suppressing malignancies proliferation. Ferulic acid can also induce apoptosis in cancer cells and thus may be applied as an effective therapeutic agent. When combined with cytostatics, FA enhances the efficacy of chemotherapy and mitigates its adverse effects.

**Figure 7:**
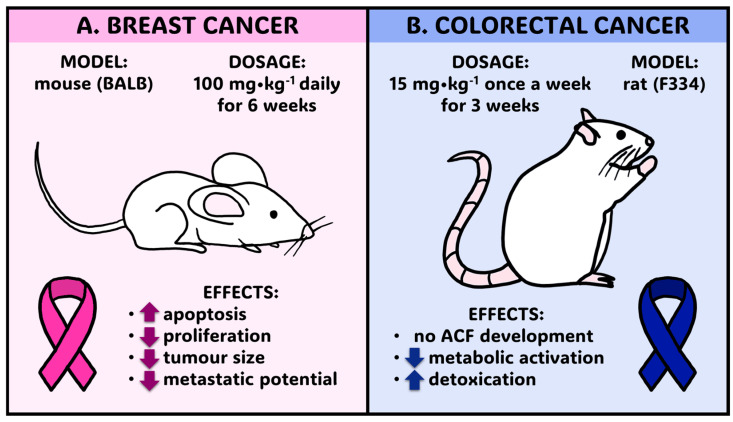
A summary of in vivo studies of ferulic acid application in cancer treatment—FA dosage and its resulting effects on cancer cells: (**A**) ferulic acid administration to mice for breast cancer treatment; (**B**) ferulic acid administration to rats for colorectal cancer treatment. Arrow heading up—enhanced effect; arrow heading down—diminished effect. The figures were prepared according to the information provided by (**A**) Zhang et al. (2016) [2] and (**B**) Kawabata et al. 2000 [38].

On the other hand, one of the major limitations of the broader application of ferulic acid in cancer treatment is its poor bioavailability, due to its low absorption rate in the gut, rapid metabolism, and clearance from the body [43]. This feature significantly limits its therapeutic efficacy. To overcome this constraint, several novel FA delivery methods have been proposed, including application of nanoformulations (e.g., nanoparticles) or polymer-based delivery systems [44]. Ferulic acid absorption and bioactivity might be also improved with the use of its derivatives, such as FA esters. Moreover, it has a relatively short half-life and consequently needs to be administered frequently or as a part of an advanced drug formulation which sustains its action. Last but not least, ferulic acid research on human cancer treatment still lacks large-scale clinical trials to confirm its safety and efficacy. Even data on in vivo studies on animals are highly limited (Figure 7). Further studies on FA interactions with various anticancer drugs are needed. The pros and cons of ferulic acid application in cancer treatment are listed in Table 2.

## 7. Developments and Insights of Ferulic Acid Sources and Its Application in Cancer Treatment

In the near future, in addition to clinical trials, which appear to be of the highest importance, the main focus should also be directed towards the extraction of FA from various sources. Generally, this can be achieved through the degradation of biomass using biotechnological methods. Such investigations should involve the selection of an appropriate biomass pretreatment method, lignin decomposition into polyphenols, and their subsequent separation and purification. The possibility of extracting FA from other sources, typically regarded as waste such as lignin generated during lignocellulose processing into simple sugars, may become an attractive alternative compared to traditional methods of FA extraction. Simple sugars are primarily utilized for second-generation biofuels, whereas lignin has so far been considered merely as waste. We anticipate that such waste can be utilized to produce various polyphenols, including FA, with increasing yields in the next few years.

Considering cancer therapy, ferulic acid has been explored in various in vitro and in vivo studies. Nevertheless, there are still many aspects that can be improved in that matter. One such factor is enhancement of ferulic acid bioavailability, which might be achieved by the formulation of highly advanced delivery systems, based on novel innovative materials, nanoparticles, or biopolymers enabling FA sustained release. Moreover, its long-term safety and side effects on humans, particularly when administered with standard anticancer therapy, must be tested. The role of FA in the prevention of recurrence and reduction of resistance to chemotherapy also needs to be analyzed. There is no precise data confirming the effectiveness of ferulic acid application in various types of cancers and stages of the disease. Additionally, personalized treatment approaches, in which individual patients’ responses to ferulic acid are determined, might be considered while optimizing its use in cancer therapy.

## 8. Conclusions

Ferulic acid (FA) has many anticancer activities, including inhibiting proliferation, invasion, metastasis, and angiogenesis of malignant tumors. FA participates in modulation of cell proteins and enzyme activity, gene expression, and signaling pathways. It also shows synergy with other antioxidants, e.g., vitamin E derivatives or resveratrol, as well as with standard cytostatic agents used in cancer treatment, to improve efficacy and reduce side effects. We have shown in this review that FA has positive effects on the most common cancers. FA may potentially act as a novel synergic agent when used together with standard therapy, particularly in nanoformulations. Future clinical trials will show whether FA plays the expected complementary role to standard cytostatic therapy or nanotherapy for malignancies.

## Figures and Tables

**Table 2:** Pros and cons of ferulic acid application in cancer treatment.

Pros	Cons
Inducing apoptosis of cancer cells	Low bioavailability for humans
Limiting cancer spread and metastasis	Short half-life
Providing additional beneficial for human health (e.g., antioxidant and anti-inflammatory properties)	A vast majority of studies have been conducted on cell lines (in vitro).
Reducing the risk of mutations leading to cancer	Highly limited in vivo studies (mainly on rodents)
Modulating oncogenic signaling	Lack of large-scale clinical trials
Increasing the efficacy of standard treatment (e.g., chemotherapy)	Efficacy of its action differs and depends on cancer type.
Exhibiting low or no toxicity for normal cells (while administrated at therapeutic doses)	Its application in humans might require personalized treatment approaches.
Showing synergy with other antioxidants	Lack of standardized dosage
It might be applied in various chemical formulas—in the form of a derivative (e.g., esters).	It needs to be administrated frequently or as a part of an advanced drug formulation.
Compatible with a broad range of delivery materials (e.g., nanoparticles and biopolymers)	No data on long-term use
It can be obtained from renewable sources.	Possible interaction with drugs/medicines
Wide availability of sources from which it can be extracted	It might cause an allergic reaction
Application in formulations, such as nanoparticles or biopolymers, enhances accuracy of tumor targeting.	Its derivatives have higher bioavailability.

## Data Availability

No new data were created or analyzed in this study.

## References

[1] Shen N., Wang T., Gan Q., Liu S., Wang L., Jin B. (2022). Plant flavonoids: Classification, distribution, biosynthesis, and antioxidant activity. Food Chem..

[2] Zhang X., Lin D., Jiang R., Li H., Wan J., Li H. (2016). Ferulic acid exerts antitumor activity and inhibits metastasis in breast cancer cells by regulating epithelial to mesenchymal transition. Oncol. Rep..

[3] Babbar R., Dhiman S., Grover R., Kaur A., Arora S. (2021). Comprehensive Review on Therapeutic Applications of Ferulic Acid and its Novel Analogues: A Brief Literature. Mini Rev. Med. Chem..

[4] Zhang L., Han Z., Granato D. (2021). Polyphenols in foods: Classification, methods of identification, and nutritional aspects in human health. Adv. Food Nutr. Res..

[5] Harms L.M., Scalbert A., Zamora-Ros R., Rinaldi S., Jenab M., Murpy N., Achaintre D., Jønneland A., Olsen A., Overvad K. (2020). Plasma polyphenols associated with lower high-sensitivity C-reactive protein concentrations: A cross-sectional study within the European Prospective Investigation into Cancer and Nutrition. Br. J. Nutr..

[6] Gupta A., Sing A.K., Loka M., Pandey A.K., Bishayee A. (2021). Ferulic acid-mediated modulation of apoptotic signaling pathways in cancer. Adv. Protein Chem. Struct. Biol..

[7] Airoldi C., La Ferla B., Orazio G.D., Ciaramelli C., Palmioli A. (2018). Flavonoids in the Treatment of Alzheimer’s and Other Neurodegenerative Diseases. Curr. Med. Chem..

[8] Cavalcanti G.R., Duarte F.C., Converti A., de Lima A.A.N. (2021). Ferulic Acid Activity in Topical Formulations: Technological and Scientific Prospecting. Curr. Pharm. Des..

[9] Roszkowski S. (2023). Application of Polyphenols and Flavonoids in Oncological Therapy. Molecules.

[10] Bugg T.D.H., Rahmanpour R. (2015). Enzymatic conversion of lignin into renewable. Curr. Opin. Chem. Biol..

[11] Chauhan P.S. (2020). Role of various bacterial enzymes in complete depolymerization of lignin. A Rev. Biocatal. Agric. Biotechnol..

[12] Iram A., Cekmecelioglu D., Demirci A. (2022). Integrating 1G with 2G Bioethanol Production by Using Distillers’ Dried Grains with Solubles (DDGS) as the Feedstock for Lignocellulolytic Enzyme Production. Fermentation.

[13] Bray F., Laversanne M., Sung H., Ferlay J., Siegel R.L., Soerjomataram I., Jemal A. (2024). Global Cancer Statistics 2024: GLOBOCAN Estimates of Incidence and Mortality Worldwide for 36 Cancers in 185 Countries. CA A Cancer J. Clin..

[14] Siegel R.L., Giaquinto A.N., Jemal A. (2024). Cancer statistics. CA Cancer J. Clin..

[15] Bao X., Li W., Jia R., Meng D., Zhang H., Xia L. (2023). Molecular mechanism of ferulic acid and its derivatives in tumor progression. Pharmacol. Rep..

[16] Tuli H.S., Kumar A., Ramniwas S., Coundhary R., Aggarwal D., Kumar M., Ujjawal S., Parashar N.C., Haque S., Sak K. (2022). Ferulic Acid: A Natural Phenol That Inhibits Neoplastic Events through Modulation of Oncogenic Signaling. Molecules.

[17] Damasceno S.S., Dantas B.B., Ribeiro-Filho J. (2017). Chemical Properties of Caffeic and Ferulic Acids in Biological System: Implications in Cancer Therapy. A Review, Curr. Pharm. Des..

[18] Yang G.W., Jiang J.S., Lu W.Q. (2015). Ferulic Acid Exerts Anti-Angiogenic and Anti-Tumor Activity by Targeting Fibroblast Growth Factor Receptor 1-Mediated Angiogenesis. Int. J. Mol. Sci..

[19] Paciello F., Fetoni A.R., Mezzogori D., Rolesi R., Di Pino A., Paludetti G., Grassi C., Troiani D. (2020). The dual role of curcumin and ferulic acid in counteracting chemoresistance and cisplatin-induced ototoxicity. Sci. Rep..

[20] Zheng Y., You X., Chen L., Huang J., Wang L., Wu J., Guan S. (2019). Biotherapeutic Nanoparticles of Poly (Ferulic Acid) Delivering Doxorubicin for Cancer Therapy. J. Biomed. Nanotechnol..

[21] Sarwar T., Zafaryab M., Husain M.A., Ishqi H.M., Rehman S.U., Rizvi M.M.A., Tabish M. (2015). Redox cycling of endogenous copper by ferulic acid leads to cellular DNA breakage and consequent cell death: A putative cancer chemotherapy mechanism. Toxicol. Appl. Pharmacol..

[22] Mancuso C., Santangelo R. (2014). Ferulic acid: Pharmacological and toxicological aspects. Food Chem. Toxicol..

[23] Arnold M., Morgan E., Rumgay H., Mafra A., Singh D., Laversanne M., Vigant J., Gralow J.R., Cardoso F., Siesling S. (2022). Current and future burden of breast cancer: Global statistics for 2020 and 2040. Breast.

[24] Predarska I., Saoud M., Drača D., Morgan I., Komazec T., Eichhorn T., Michaliović E., Dunderović D., Mijatović S., Mihajlović-Ivanić D. (2022). Mesoporous Silica Nanoparticles Enhance the Anticancer Efficacy of Platinum (IV)-Phenolate Conjugates in Breast Cancer Cell Lines. Nanomaterials.

[25] Jiang H., Li M., Du K., Ma C., Cheng Y., Wang S., Nie X., Fu C., He Y. (2021). Traditional Chinese Medicine for adjuvant treatment of breast cancer: Taohong Siwu Decoction. Chin. Med..

[26] Alotaibi B., El-Masry T.A., Elekhnawy E., Mokhatar F.A., El-Seadawy H.M., Negm W. (2024). Studying the effects of secondary metabolites isolated from Cycas thouarsii R.Br. leaves on MDA-MB-231 breast cancer cells. Artif. Cells Nanomed. Biotechnol..

[27] de Freitas Meirelles L.E., de Souza M.V.F., Carobeli L.R., Morelli F., Mari N.L., Damke E., Mesquita C.S.S., Teixeira J.J.V., Consolaro M.E.L., de Silva V.R.S. (2023). Combination of Conventional Drugs with Biocompounds Derived from Cinnamic Acid: A Promising Option for Breast Cancer Therapy. Biomedicines.

[28] Sudhagar S., Sathya S., Anuradha R., Gokulapirya G., Geetharani Y., Lakshmi B.S. (2018). Inhibition of epidermal growth factor receptor by ferulic acid and 4-vinylguaiacol in human breast cancer cells. Biotechnol. Lett..

[29] Rezaei A., Varshosaz J., Fesharaki M., Farhang A., Jafari S.M. (2019). Improving the solubility and in vitro cytotoxicity (anticancer activity) of ferulic acid by loading it into cyclodextrin nanosponges. Int. J. Nanomed..

[30] Helmy S.A., El-Mofty S., El Gaya A.M., El-Sherbiny I.M., El-Far Y.M. (2022). Novel doxorubicin/folate-targeted trans-ferulic acid-loaded PLGA nanoparticles combination: In-vivo superiority over standard chemotherapeutic regimen for breast cancer treatment. Biomed. Pharmacother..

[31] AbouAitah K., Abdelaziz A.M., Higazy I.M. (2024). Functionalized carbon nanotubes for delivery of ferulic acid and diosgenin anticancer natural agents. ACS Appl. Bio Mater..

[32] Luo L., Zhu S., Tong Y., Peng S. (2020). Ferulic Acid Induces Apoptosis of HeLa and Caski Cervical Carcinoma Cells by Down-Regulating the Phosphatidylinositol 3-Kinase (PI3K)/Akt Signaling Pathway. Med. Sci. Monit..

[33] Gao J., Yu H., Guo W., Gu L., Li Q., Yang S., Zhang Y., Wang Y. (2018). The anticancer effects of ferulic acid is associated with induction of cell cycle arrest and autophagy in cervical cancer cells. Cancer Cell Int..

[34] Wang D., Guo D., Tang Y., Qi M., Fang J., Zhang Y., Chai Y., Cao Y., Lv D. (2022). A multi-omics study of the anti-cancer effect of a ferulic acid derivative FA-30. Mol. Omics.

[35] Russo G.J., Campisi D., Di Mauro M., Regis F., Reale G., Marranzano M., Ragusa R., Solinas T., Madonia M., Cimino S. (2017). Dietary Consumption of Phenolic Acids and Prostate Cancer: A Case-Control Study in Sicily. S. Italy Mol..

[36] Eroğlu C., Seçme M., Bağcı G., Dodurga Y. (2015). Assessment of the anticancer mechanism of ferulic acid via cell cycle and apoptotic pathways in human prostate cancer cell lines. Tumour Biol..

[37] Eitsuka T., Tatewaki N., Nishida H., Kurata T., Nakagawa K., Miyazawa T. (2014). Synergistic inhibition of cancer cell proliferation with a combination of δ-tocotrienol and ferulic acid. Biochem. Biophys. Res. Commun..

[38] Kawabata K., Yamamoto T., Hara A., Shimizu M., Yamada Y., Matsunaga K., Tanaka T., Mori H. (2000). Modifying effects of ferulicacid on azoxymethane-induced colon carcinogenesis in F344 rats. Cancer Lett..

[39] Janicke B., Onning G., Oredsson S.M. (2005). Differential effects of ferulic acid and p-coumaric acid on S phase distribution and length of S phase in the human colonic cell line Caco-2. J. Agric. Food Chem..

[40] Roy N., Narayanankutty A., Nazeem P.A., Valslan R., Babu T.D., Mathew D. (2016). Plant Phenolics Ferulic Acid and P-Coumaric Acid Inhibit Colorectal Cancer Cell Proliferation through EGFR Down Regulation. Asian Pac. J. Cancer Prev..

[41] de Souza Rosa L., Jordão N.A., da Costa Pereira Soarez N., de Mesquita J.F., Monterio M., Teodoro A.J. (2018). Pharmacokinetic, antiproliferative and apoptotic effects of phenolic acids in human colon adenocarcinoma cells using in vitro and in silico approaches. Mol. A J. Synth. Chem. Nat. Prod. Chem..

[42] Sawata Y., Matsukawa T., Doi S., Tsunoda T., Arikawa N., Matsunaga N., Ohnuki K., Shirasawa S., Kotake Y. (2019). A novel compound, ferulic acid-bound resveratrol, induces the tumor suppressor gene p15 and inhibits the three-dimensional proliferation of colorectal cancer cells. Mol. Cell Biochem..

[43] Pyrzyńska K. (2024). Ferulic acid—A brief review of its extraction, bioavailability and biological activity. Separations.

[44] Purushothaman J.R., Rizwanullah M. (2024). Ferulic Acid: Comprehensive Review. Cureues.

